# Supplementary material for: Molecular basis for the adaptive evolution of environment-sensing by H-NS proteins
Source: eLife. 2021 Jan 7;10:e57467. doi: 10.7554/eLife.57467 (PMC7817174; doi:10.7554/eLife.57467)
Supplement: Supplementary file 1. [file elife-57467-supp1.docx]

Supplementary Information

**Molecular Basis for the Adaptive Evolution of Environment Sensing by H-NS Proteins**

Xiaochuan Zhao,^a#^ Umar F. Shahul Hameed,^b#^ Vladlena Kharchenko,^c#^ Chenyi Liao,^a^ Franceline Huser,^c^ Jacob M. Remington,^c^ Anand K. Radhakrishnan,^c^ Mariusz Jaremko,^b^ Łukasz Jaremko,^b^* Stefan T. Arold,^c,d^* Jianing Li^a^*

a. Department of Chemistry, The University of Vermont, Burlington, VT, USA 05405.

b. King Abdullah University of Science and Technology (KAUST), Computational Bioscience Research Center (CBRC), Biological and Environmental Science and Engineering (BESE), Thuwal, 23955-6900, Saudi Arabia

c. King Abdullah University of Science and Technology (KAUST), Biological and Environmental Science and Engineering (BESE), Thuwal, 23955-6900, Saudi Arabia

d. Centre de Biochimie Structurale, CNRS, INSERM, Université de Montpellier, 34090 Montpellier, France

# contributed equally

*** correspondence to:** JL ([jianing.li@uvm.edu](mailto:jianing.li@uvm.edu)), STA ([stefan.arold@kaust.edu.sa](mailto:stefan.arold@kaust.edu.sa)) or LJ ([lukasz.jaremko@kaust.edu.sa](mailto:lukasz.jaremko@kaust.edu.sa))

Includes:

1. Supplementary file 1A – 1F
2. Supplementary References

**Supplementary file 1A**. The sequence similarity matrix of H-NS_ST_, H-NS_EA_, H-NS_BA_, and H-NS_IL_

|  | H-NS_ST_ | H-NS_EA_ | H-NS_BA_ | H-NS_IL_ |
| --- | --- | --- | --- | --- |
| H-NS_ST_ | 100 | 89.55 | 62.22 | 40.74 |
| H-NS_EA_ | 89.55 | 100 | 62.69 | 39.85 |
| H-NS_BA_ | 62.22 | 62.69 | 100 | 33.58 |
| H-NS_IL_ | 40.74 | 39.85 | 33.58 | 100 |

**Supplementary file 1B.** Summary of reported H-NS simulations (MD = unbiased molecular dynamics simulation; US = umbrella sampling simulations). The CHARMM36 force field (1) with TIP3P water model was used. Total simulation length = 5.7 μs.

| Simulation | Num. of atoms | Box  (nm^3^) | [NaCl]  (mol/L) | T(K) | Time (ns) | Note |  |
| --- | --- | --- | --- | --- | --- | --- | --- |
| **H-NS_ST_ tetramer** | | | | | | Each tetramer model (illustrated in **Fig. 1** of the main text) contains two full-length H-NS monomers (PDB templates: 3NR7 and 2L93) and two partial monomers of site1, to mimic the full-length H-NS monomers in the polymeric state. Each construct was simulated with 2 replicas. |  |
| MD | 102,953 | 13⋅9⋅10 | 0.15 | 293 | 200×2 |  |  |
| MD | 102,101 | 13⋅9⋅10 | 0.50 | 293 | 200×2 |  |  |
| MD | 102,953 | 13⋅9⋅10 | 0.15 | 313 | 200×2 |  |  |
| **H-N_EA_ tetramer** | | | | | |  |  |
| MD | 100,055 | 13⋅9⋅10 | 0.15 | 293 | 200×2 |  |  |
| MD | 99,227 | 13⋅9⋅10 | 0.50 | 293 | 200×2 |  |  |
| MD | 100,055 | 13⋅9⋅10 | 0.15 | 313 | 200×2 |  |  |
| **H-NS_BA_ tetramer** | | | | | |  |  |
| MD | 104,551 | 13⋅9⋅10 | 0.15 | 293 | 200×2 |  |  |
| MD | 103,691 | 13⋅9⋅10 | 0.50 | 293 | 200×2 |  |  |
| MD | 104,551 | 13⋅9⋅10 | 0.15 | 313 | 200×2 |  |  |
| **H-NS_IL_ tetramer** | | | | | |  |  |
| MD | 104,733 | 13⋅9⋅10 | 0.15 | 293 | 200×2 |  |  |
| MD | 103,896 | 13⋅9⋅10 | 0.50 | 293 | 200×2 |  |  |
| MD | 104,733 | 13⋅9⋅10 | 0.15 | 313 | 200×2 |  |  |
| **H-NS_ST_ site2 dimer** | | | | | |  |  |
| US | 45,920 | 13⋅6⋅6 | 0.15 | 293 | 54×80 | Each site2 dimer model contains two site2 monomers (residues 50-82). We used 80 windows (at an interval of ~0.3 Å) to sample the pathway of dimer dissociation along the direction of increasing center of mass (COM) distance at the constraint of 1.2  kcal/mol/Å^2^, each umbrella window was simulated for 54 ns. |  |
| US | 45,524 | 13⋅6⋅6 | 0.50 | 293 | 54×80 |  |  |
| US | 45,920 | 13⋅6⋅6 | 0.15 | 313 | 54×80 |  |  |
| **H-N_EA_ site2 dimer** | | | | | |  |  |
| US | 45,863 | 13⋅6⋅6 | 0.15 | 293 | 54×80 |  |  |
| US | 45,467 | 13⋅6⋅6 | 0.50 | 293 | 54×80 |  |  |
| US | 45,863 | 13⋅6⋅6 | 0.15 | 313 | 54×80 |  |  |
| **H-NS_BA_ site2 dimer** | | | | | |  |  |
| US | 45,968 | 13⋅6⋅6 | 0.15 | 293 | 54×80 |  |  |
| US | 45,572 | 13⋅6⋅6 | 0.50 | 293 | 54×80 |  |  |
| US | 45,968 | 13⋅6⋅6 | 0.15 | 313 | 54×80 |  |  |
| **H-NS_IL_ site2 dimer** | | | | | |  |  |
| US | 45,899 | 13⋅6⋅6 | 0.15 | 293 | 54×80 |  |  |
| US | 45,503 | 13⋅6⋅6 | 0.50 | 293 | 54×80 |  |  |
| US | 45,899 | 13⋅6⋅6 | 0.15 | 313 | 54×80 |  |  |

**Computational Data analysis:** All the data analyses were carried out in GROMACS, VMD Tcl scripts, and in-house Python programs. In particular, root-mean-square fluctuations (RMSF), root-mean-square deviation (RMSD), salt-bridge interaction, polar interaction, and hydrophobic interaction were analyzed in VMD (2). For salt-bridges, the O and N atoms in the charged residues (Arg, His, Lys, Asp, and Glu) were used with a distance cut-off of 4.5 Å. For the polar interaction, the atoms in the side chains with the partial charge cutoff (> 0.3 unit for a polar contact) were used with a distance cutoff of 4.5 Å. The cut-off for classifying hydrophobic interactions was 6.0 Å between the C atoms of the hydrophobic residues. The relative percentage (*P*) of hydrophobic contacts is defined as **Eq. 1**.

$P=\sum_{n=1}^{N} \frac{C_{n}}{NM}$ (Eq. 1)

N is the total number of frames, *C_n_* is the number of hydrophobic contacts at frame *n*, and *M* is the largest value in the series of *C_n_*. The PMFs were determined using the Weighted Histogram Analysis Method (WHAM) (3) implemented in GROMACS. All the visualization was performed with python, VMD, Pymol (Schrödinger, Inc.), and Maestro (Schrödinger, Inc.).

**Supplementary file 1C.** The average RMSF of each helical region in H-NS site1/site2 at different conditions. The average of the last 10 ns out of a total of 200 ns of both replicas were used.

|  | [NaCl] (mol/L) | T(K) | | α1 (Å) | α2 (Å) | α3N (Å) | α3C (Å) | α4 (Å) |
| --- | --- | --- | --- | --- | --- | --- | --- | --- |
| **H-NS_ST_** | | | | | | | | |
| 1 | 0.15 | | 293 | 3.5 ± 1.2 | 2.3 ± 0.4 | 4.5 ± 1.0 | 3.3 ± 0.9 | 5.1 ± 1.8 |
| 2 | 0.50 | | 293 | 3.3 ± 1.3 | 2.3 ± 0.3 | 4.6 ± 1.3 | 4.7 ± 1.3 | 5.8 ± 1.7 |
| 3 | 0.15 | | 313 | 3.7 ± 1.5 | 2.4 ± 0.3 | 4.9 ± 1.2 | 3.9 ± 0.8 | 5.0 ± 1.3 |
| **H-NS_EA_** | | | | | | | | |
| 4 | 0.15 | | 293 | 4.7 ± 2.0 | 2.3 ± 0.2 | 4.3 ± 1.1 | 4.0 ± 1.9 | 14.6 ± 4.3 |
| 5 | 0.50 | | 293 | 6.2 ± 2.3 | 2.4 ± 0.3 | 4.7 ± 1.2 | 5.3 ± 1.9 | 14.6 ± 4.7 |
| 6 | 0.15 | | 313 | 5.3 ± 1.8 | 2.6 ± 0.3 | 5.0 ± 1.3 | 4.7 ± 1.3 | 15.7 ± 3.1 |
| **H-NS_BA_** | | | | | | | | |
| 7 | 0.15 | | 293 | 2.6 ± 0.8 | 2.4 ± 0.5 | 5.7 ± 3.1 | 4.5 ± 1.9 | 9.1 ± 4.9 |
| 8 | 0.50 | | 293 | 2.7 ± 0.9 | 2.3 ± 0.3 | 5.4 ± 1.7 | 4.4 ± 1.2 | 8.5 ± 5.9 |
| 9 | 0.15 | | 313 | 2.5 ± 0.8 | 2.6 ± 0.4 | 4.9 ± 1.4 | 5.3 ± 1.9 | 6.1 ± 1.7 |
| **H-NS_IL_** | | | | | | | | |
| 10 | 0.15 | | 293 | 1.5 ± 0.4 | 2.2 ± 0.2 | 4.6 ± 1.9 | 4.7 ± 1.7 | 15.8 ± 7.3 |
| 11 | 0.50 | | 293 | 1.5 ± 0.3 | 2.3 ± 0.2 | 5.1 ± 1.4 | 4.2 ± 1.2 | 8.1 ± 2.4 |
| 12 | 0.15 | | 313 | 1.6 ± 0.3 | 2.5 ± 0.3 | 5.7 ± 1.8 | 4.2 ± 1.0 | 10.2 ± 2.9 |

**Supplementary file 1D**. Free energy prediction of mutations by prediction tools

| Site 1 Mutations | Maestro | PremPS |
| --- | --- | --- |
|  | ΔΔG(kcal/mol) | ΔΔG (kcal/mol) |
|  |  |  |
| L5A | 1.20 | 0.81 |
| L5K | 1.04 | 0.7 |
| L5R | 1.01 | 0.75 |
| L5E | 0.82 | 1.0 |
| L5D | 1.24 | 1.01 |
| L5Q | 0.71 | 0.86 |
| L5N | 1.21 | 0.89 |
|  |  |  |
| L8A | 1.16 | 1.03 |
| L8K | 1.04 | 1.53 |
| L8R | 1.22 | 1.5 |
| L8E | 0.68 | 1.65 |
| L8D | 1.69 | 1.72 |
| L8Q | 0.70 | 1.3 |
| L8N | 1.43 | 1.72 |
|  |  |  |
| I11A | 0.39 | 0.81 |
| I11K | 0.86 | 0.69 |
| I11R | 1.03 | 0.99 |
| I11E | -0.13 | 1.15 |
| I11D | 0.04 | 1.11 |
| I11Q | 0.20 | 0.9 |
| I11N | 0.25 | 1.11 |
|  |  |  |
| R12A | -0.01 | -0.12 |
| R12K | 0.18 | -0.09 |
| R12R | - | - |
| R12E | -0.31 | -0.14 |
| R12D | -0.21 | -0.05 |
| R12Q | -0.14 | -0.15 |
| R12N | -0.05 | -0.02 |
|  |  |  |
| L14A | 1.26 | 0.84 |
| L14K | 1.28 | 1.24 |
| L14R | 1.22 | 0.97 |
| L14E | 0.93 | 1.67 |
| L14D | 2.17 | 1.68 |
| L14Q | 0.65 | 1.25 |
| L14N | 1.70 | 1.66 |
|  |  |  |
| L23A | 0.53 | 1.18 |
| L23K | 1.23 | 1.35 |
| L23R | 1.11 | 1.33 |
| L23E | -0.03 | 1.51 |
| L23D | 0.10 | 1.68 |
| L23Q | 0.17 | 1.31 |
| L23N | 0.50 | 1.44 |
|  |  |  |
| L26A | 1.63 | 2.16 |
| L26K | 1.82 | 2.34 |
| L26R | 1.31 | 2.32 |
| L26E | 1.58 | 2.41 |
| L26D | 2.24 | 2.53 |
| L26Q | 0.73 | 2.28 |
| L26N | 2.35 | 2.51 |
|  |  |  |
| E28A | 1.03 | 0.25 |
| E28K | 2.00 | 0.31 |
| E28R | 1.62 | 0.33 |
| E28E | - | - |
| E28D | 0.07 | 0.54 |
| E28Q | 0.36 | 0.37 |
| E28N | 0.75 | 0.35 |
|  |  |  |
| L30A | 0.69 | 1.12 |
| L30K | 1.12 | 1.16 |
| L30R | 1.31 | 1.00 |
| L30E | 0.40 | 1.39 |
| L30D | 1.28 | 1.59 |
| L30Q | 0.33 | 1.04 |
| L30N | 0.75 | 1.35 |
|  |  |  |
| L33A | 1.80 | 2.03 |
| L33K | 2.06 | 2.19 |
| L33R | 1.65 | 2.19 |
| L33E | 2.13 | 2.28 |
| L33D | 2.80 | 2.45 |
| L33Q | 1.30 | 2.18 |
| L33N | 2.89 | 2.37 |
|  |  |  |
| V36A | 0.77 | 1.69 |
| V36K | 0.92 | 2.3 |
| V36R | 0.89 | 2.25 |
| V36E | 0.17 | 2.32 |
| V36D | 1.27 | 2.45 |
| V36Q | 0.60 | 2.31 |
| V36N | 0.95 | 2.36 |
|  |  |  |
| E39A | 1.04 | 0.36 |
| E39K | 2.16 | 0.43 |
| E39R | 1.53 | 0.49 |
| E39E | - | - |
| E39D | 0.16 | 0.43 |
| E39Q | 0.09 | 0.44 |
| E39N | 0.78 | 0.48 |

**Supplementary file 1E.** Comparison of prediction tools and free energy calculations

| Site 1  Mutations | Maestro | PremPS | Free Energy Calculations |
| --- | --- | --- | --- |
|  | ΔΔG(kcal/mol) | ΔΔG (kcal/mol) | ΔΔG(kcal/mol) |
| L23K | 1.23 | 1.35 | 12.1 |
| L23R | 1.11 | 1.33 | 14.5 |
| L23E | -0.03 | 1.51 | 2.6 |
| L23D | 0.10 | 1.68 | 4.3 |
| L23Q | 0.17 | 1.31 | 4.6 |
| L23N | 0.50 | 1.44 | 2.6 |
| L26A | 1.63 | 2.16 | 8.42 |
| L26K | 1.82 | 2.34 | 6.56 |
| L26R | 1.31 | 2.32 | 6.88 |
| L26E | 1.58 | 2.41 | 7.92 |
| L26D | 2.24 | 2.53 | 0.58 |
| L26Q | 0.73 | 2.28 | 10.56 |
| L26N | 2.35 | 2.51 | 1.93 |

**Supplementary file 1F.** Statistics of conservative charged contacts in MD simulations (sidechain N-O distances in Å, averaged over the last 50 ns of two simulation replicas).

|  | 293 K, 0.15 M NaCl | 313 K, 0.15 M NaCl | 293 K, 0.50 M NaCl |
| --- | --- | --- | --- |
| **E52-R56** |  |  |  |
| H-NS_ST_ | 7.2 ± 2.7 | 7.5 ± 3.0 | 7.0 ± 2.8 |
| H-NS_EA_ | 6.9 ± 2.8 | 7.3 ± 2.9 | 7.5 ± 3.0 |
| H-NS_BA_ | N/A due to E52Q | | |
| H-NS_IL_ | N/A due to E52A | | |
| **R/K54/53-D71’** |  |  |  |
| H-NS_ST_ | 7.4 ± 1.9 | 8.1 ± 1.9 | 10.3 ± 2.2 |
| H-NS_EA_ | 9.2 ± 1.8 | 8.9 ± 2.3 | 8.7 ± 2.1 |
| H-NS_BA_ | N/A due to D71N  N/A due to R54Q | | |
| H-NS_IL_ |  |  |  |
| **R/K54/53-E74’** |  |  |  |
| H-NS_ST_ | 4.6 ± 1.2 | 7.9 ± 4.1 | 7.8 ± 3.2 |
| H-NS_EA_ | 6.9 ± 3.8 | 9.1 ± 4.4 | 5.0 ± 3.3 |
| H-NS_BA_ | 8.5 ± 4.8 | 5.6 ± 2.2 | 8.4 ± 2.5 |
| H-NS_IL_ | 5.2 ± 2.0 | 8.4 ± 5.2 | 8.4 ± 3.52 |
| **K57/56-D67’/68’** |  |  |  |
| H-NS_ST_ | 3.9 ± 1.3 | 3.7 ± 1.1 | 4.1 ± 1.8 |
| H-NS_EA_ | 3.8 ± 1.4 | 4.3 ± 1.5 | 3.6 ± 1.3 |
| H-NS_BA_ | 4.9 ± 2.9 | 3.5 ± 1.1 | 3.5 ± 0.9 |
| H-NS_IL_ |  | N/A due to D57I |  |
| **R62-E/D63** |  |  |  |
| H-NS_ST_ | 10.0 ± 2.3 | 7.0 ± 2.4 | 9.8 ± 2.1 |
| H-NS_EA_ | 8.7 ± 2.0 | 8.6 ± 2.2 | 9.6 ± 2.1 |
| H-NS_BA_ | 9.7 ± 1.8 | 7.1 ± 2.9 | 7.9 ± 2.5 |
| H-NS_IL_ | N/A due to E62S | | |

**References**

1. Best, R.B., Zhu, X., Shim, J., Lopes, P.E., Mittal, J., Feig, M. and MacKerell Jr, A.D. (2012) Optimization of the additive CHARMM all-atom protein force field targeting improved sampling of the backbone ϕ, ψ and side-chain χ1 and χ2 dihedral angles. *Journal of chemical theory and computation*, **8**, 3257-3273.

2. Humphrey, W., Dalke, A. and Schulten, K. (1996) VMD: visual molecular dynamics. *Journal of molecular graphics*, **14**, 33-38.

3. Hub, J.S., De Groot, B.L. and Van Der Spoel, D. (2010) g_wham— A Free Weighted Histogram Analysis Implementation Including Robust Error and Autocorrelation Estimates. *Journal of chemical theory and computation*, **6**, 3713-3720.
